# Mesoporous TiO_2_ Yolk-Shell Microspheres for Dye-sensitized Solar Cells with a High Efficiency Exceeding 11%

**DOI:** 10.1038/srep14178

**Published:** 2015-09-18

**Authors:** Zhao-Qian Li, Wang-Chao Chen, Fu-Ling Guo, Li-E Mo, Lin-Hua Hu, Song-Yuan Dai

**Affiliations:** 1 Key Laboratory of Novel Thin-Film Solar Cells, Institute of Applied Technology, Hefei Institutes of Physical Science, Chinese Academy of Sciences, Hefei, Anhui, 230031, P. R. China; 2 Beijing Key Laboratory of Novel Thin-Film Solar Cells, North China Electric Power University, Beijing, 102206, P. R. China

## Abstract

Yolk-shell TiO_2_ microspheres were synthesized *via* a one-pot template-free solvothermal method building on the aldol condensation reaction of acetylacetone. This unique structure shows superior light scattering ability resulting in power conversion efficiency as high as 11%. This work provided a new synthesis system for TiO_2_ microspheres from solid to hollow and a novel material platform for high performance solar cells.

Structure with an interior space always could add another excitements and interests, thus, objects with a holding capacity especially draw people’s attention. For nanomaterials, hollow micro/nanostructures are likewise fascinating because their unique structures endue them with outstanding properties, such as high surface-to-volume ratio and superior light scattering effect that make them promising for applications including lithium ion batteries^1^,^2^,^3^,^4^,^5^, catalysis^6^,^7^,^8^,^9^, chemical sensors^10^,^11^,^12^, and solar cells^13^,^14^,^15^,^16^,^17^,^18^,^19^,^20^. Among all the previously reported hollow materials, TiO_2_ hollow microspheres are of great important as the photoanode in dye-sensitized solar cells (DSSCs) due to their noticeable characteristics, e. g., high surface area for dye adsorption, low density for electrolyte diffusion and superior light scattering effect for light harvesting^14^,^16^,^17^,^18^,^21^. Up to present, many efforts have been made to improve the performance of DSSCs using TiO_2_ hollow materials as photoanode, e.g., nano-embossed hollow sphere^14^, hollow spheres^17^, multi-shell porous hollow nanoparticles^18^, and urchin-like hollow spheres^19^. The reported power conversion efficiency (PCE) of TiO_2_ hollow structure based DSSCs has reached 10.34%^14^. Nevertheless, the PCE is still lower than we expected. Therefore, exploring new strategies for synthesizing TiO_2_ hollow materials to satisfy the requirements of better performance DSSC is highly desirable.

In this work, we demonstrate a facial one-pot solvothermal approach for the synthesis of TiO_2_ microspheres based on the aldol condensation reaction in acetylacetone to eliminate water in the presence of Ti complexes. By controlling the reaction time, spheres with adjustable morphology, size and tunable interior structure from solid to yolk-shell structure was obtained. When applied as photoanode in DSSCs, the TiO_2_ yolk-shell microspheres shows superior light scattering effect and higher dye adsorption ability compared to commercial Dyesol 18 nm nanoparticles paste, leading to a high PCE value up to 11%. To our knowledge, this is the first report building on the acetylacetone condensation reaction to synthesis TiO_2_ microspheres, and 11% is yet the highest PCE value employing yolk-shell or hollow TiO_2_ microspheres as photoanode in DSSCs.

## Results and Discussion

**Figure 1:**
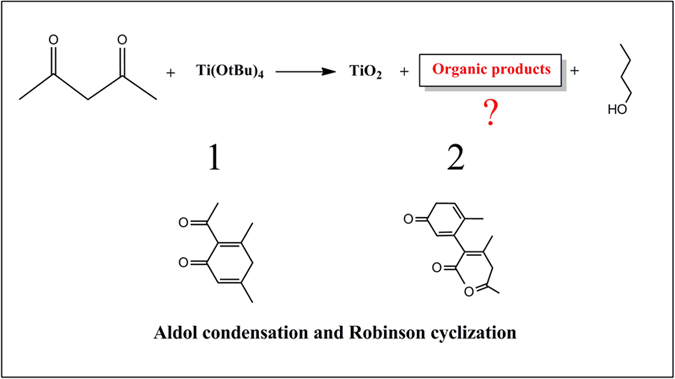
Proposed reaction mechanism. Proposed reaction leading to the formation of anatase in acetylacetone.

Yolk-shell TiO_2_ microspheres were synthesized using a one-pot solvothermal method building on the aldol condensation reaction of acetylacetone (acac). Ketones can undergo aldol condensation and eliminate water in the presence of metal complexes and are promising solvent to prepare TiO_2_ nanomaterials^22^,^23^. Whereas, interestingly, in our acac reaction system, the robinson cyclization also take place. The occurrence of aldol condensation and cyclization reactions was proved by ESI-MAS, ^13^CNMR and FTIR studies, evidenced from the formation of condensation and cyclization products and H_2_O (Fig. 1, Figure S1). Additionally, it should be noted that the addition of isopropyl alcohol is very important for the formation of TiO_2_ yolk-shell structure. Without the isopropyl alcohol, only solid spheres with a diameter range of 900–1200 nm were obtained (Figure S2).

**Figure 2:**
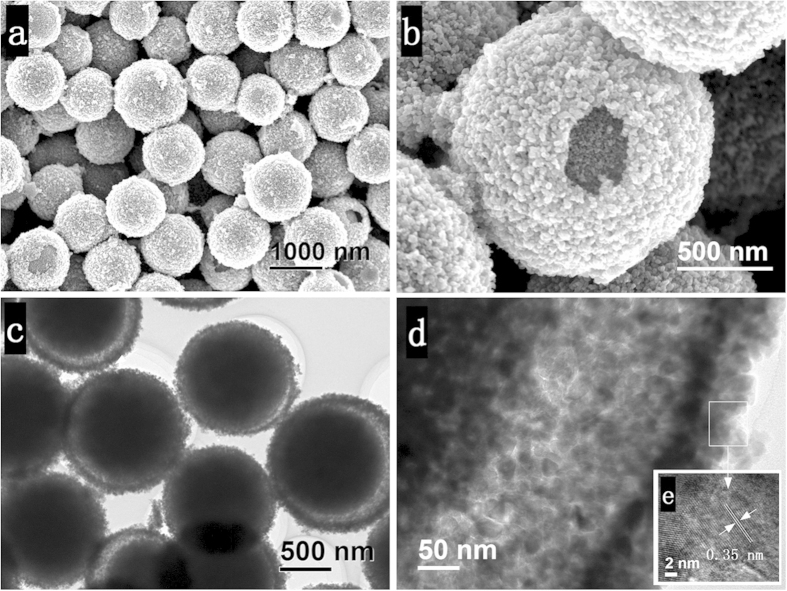
Morphology of the TiO_2_ yolk-shell structure. (**a,b**) SEM, (**c,d**) TEM and (**e**) HRTEM images of the TiO_2_ yolk-shell microspheres synthesized at 200 °C for 6 h.

Figure 2 shows the unique morphology of the as-obtained yolk-shell TiO_2_ microspheres synthesized at 200 °C for 6 h. From the scanning electron microscopy (SEM) images (Fig. 2), we can see that the TiO_2_ microspheres are well-dispersed with a rough surface and a diameter range of 1–1.4 *μ*m. The high-resolution SEM image in Fig. 2b shows that the spheres are yolk-shell structure and consists of TiO_2_ nanoparticles. The transmission electron microscopy (TEM) (Fig. 2c,d) image further displays the unique sphere-in-sphere structure with a shell thickness of ~80 nm. From Fig. 2d, it can also be seen that the yolk-shell sphere has a porous structure and are composed of TiO_2_ nanocrystals with an average diameter of ~18 nm. The high-resolution TEM image confirms the high crystalline nature of the as-obtained yolk-shell spheres (Fig. 2e).

**Figure 3:**
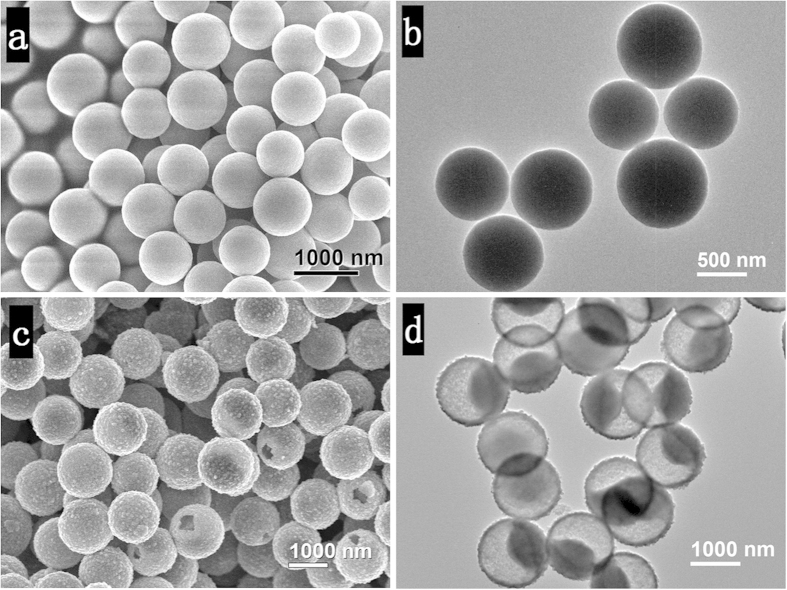
Time-dependent experiments. SEM and TEM images of the TiO_2_ microspheres synthesized at 200 °C for 4 h (**a**,**b**), and 12 h (**c**,**d**), showing transiting interior structure from solid to yolk-shell, and surface morphology from smooth to roughness.

To understand the structural evolution process of the YS-TiO_2_ microspheres, we conducted time-dependent experiments. The reaction mixture was transparent before hydrothermal treatment and no precipitation appears in the initial 2 hours reaction. After 4 h reaction, uniform, smooth, solid spheres with a diameter of about 850 nm were obtained (Fig. 3a,b). Further increase time to 6 and 12 hours (Figs 2 and 3c,d), nanoparticles consisted shells formed, leading to the formation of the unique yolk-shell structures. Interestingly, it was also found that these cores shrink with time, giving rise to an increased interspace. Furthermore, there is a growth of the outside-sphere diameter from 850 nm (4 h) to ~1.4 (6 h) and ~1.6 *μ*m (12 h).

**Figure 4:**
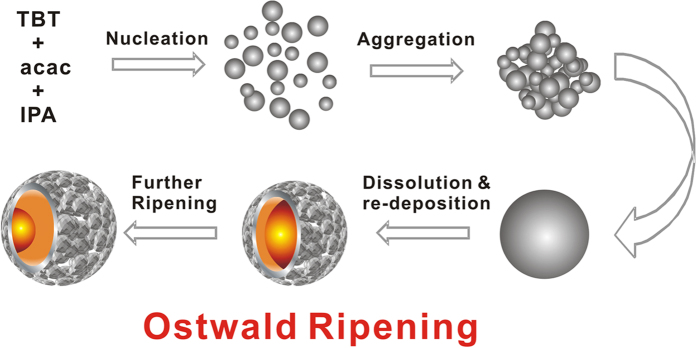
Proposed formation mechanism. Schematic formation of TiO_2_ Yolk-shell microstructures *via* Ostwald ripening process.

Based on the above investigations, the formation of such yolk-shell structural spheres might involve the nucleation, nanoparticles aggregation into spheres and subsequent Ostwald ripening process including core dissolultion and shell re-deposition^24^,^25^,^26^. As illustrated in Fig. 4, at the initial process, acac could react with Ti source to eliminate water and form the titania clusters. Then, the clusters aggregate to solid spheres. With prolonging reaction time, water is continuous generated through the aldol condensation or cyclization reaction and reacted with the TiO_2_ spheres, resulting in the dissolution and re-deposition of the surface nanoparticles, namely, the typical Ostwald ripening process. Owing to the Ostwald ripening process, surface nanoparticles formed and gradually grow into a thin sphere shell. Consequently, with the process going on, the cores gradually dissolved, leading to the novel TiO_2_ yolk-shell structure.

**Figure 5:**
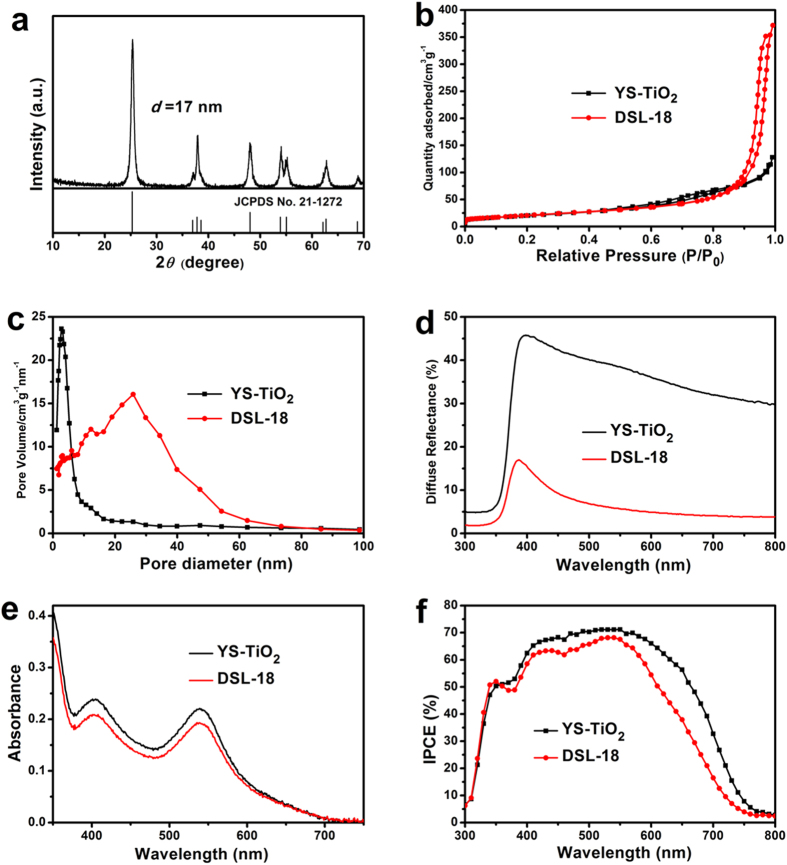
Crystal structure, BET surface area and pore size distribution of the yolk-shell TiO_2_ microspheres, diffuse reflectance and dye desorbed spectra of the YS-TiO_2_ and DSL-18 based photoanode films, IPCE of pure YS-TiO_2_ and DSL-18 based DSSC. (**a**) XRD pattern of the YS-TiO_2_; (**b**) Nitrogen adsorption/desorption isotherms and (**c**) the corresponding Barret-Joyner-Halenda (BJH) pore size distribution plots of the YS-TiO_2_ and DSL-18 after sintered; (**d**) Diffuse reflectance and (**e**) dye desorbed spectra of the anode films based on YS-TiO_2_ and DSL-18. (**e**) Incident photon-to-electron conversion efficiencies (IPCE) of the YS-TiO_2_ and DSL-18 based DSSCs. Here, the film thicknesses are 7.1 *μ*m for YS-TiO_2_, and 7.0 *μ*m for DSL-18.

The crystal structure of the YS-TiO_2_ microspheres is determined by X-ray diffraction (XRD). Figure 5a demonstrated the pure anatase TiO_2_ phase (JCPDS No. 21–1272), and the crystalline size is estimated to be about 17 nm which is close to the value observed by TEM image. The Brunauer-Emmett-Teller (BET) surface area and pore size distribution of the YS-TiO_2_ microspheres and DSL-18 were determined using nitrogen adsorption and desorption isotherms. The BET surface area of YS-TiO_2_ microspheres was 73 m^2^ g^−1^, similar with that of DSL-18 (76 m^2^ g^−1^) (Fig. 5b). The high surface area originated from the nanocrystals should facilitate dye adsorption on the TiO_2_ surface. The YS-TiO_2_ microspheres has a narrow pore size distribution, and the average pore size is 11 nm (Fig. 5c), smaller than that of DSL-18 (30 nm). This mesoporous structure could facilitate mass transport and diffusion of the electrolyte in DSSC.

To investigate the light scattering effect and dye adsorption ability of the YS-TiO_2_ microspheres, the YS-TiO_2_ microspheres and DSL-18 photoanode film with the same thicknesses were screen-printed on the FTO glass. Figure 5d shows that the YS-TiO_2_ microspheres exhibited stronger light reflectance than DSL-18 due to enhanced light scattering effect, especially in the long wavelength. In fact, the superior light scattering and harvesting ability can be evidenced by IPCE. From Fig. 5f, we can see that the YS-TiO_2_ microspheres based DSSC shows much higher IPCE than DSL-18, especially in the longer wavelength range from 570–800 nm, which should be originated from the light scattering ability^27^,^28^,^29^. Figure 5e shows the saturation adsorption of C101 dye desorbed from the YS-TiO_2_ and DSL-18 based anode film. It can be seen that, despite the YS-TiO_2_ microspheres has similar BET surface area with DSL-18, YS-TiO_2_ microspheres based photoanode film possess higher dye-loading ability than DSL-18.

**Figure 6:**
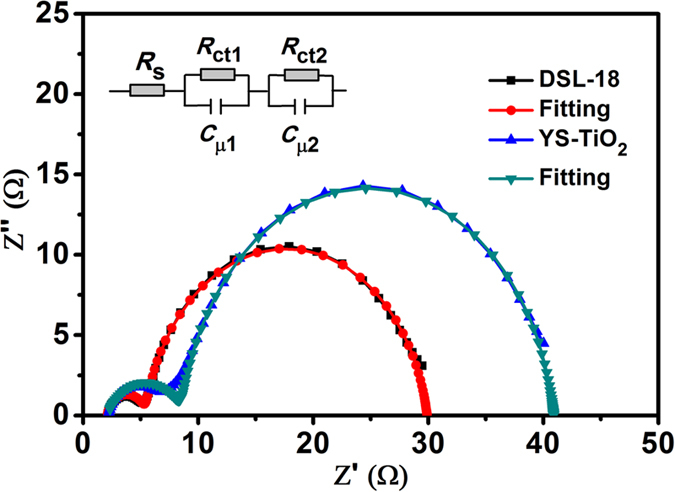
Electrochemical impedance spectroscopy (EIS) of DSSCs. (**a**) Nyquist plots of DSL-18 and YS-TiO_2_ based DSSCs measured at −0.73 V forward bias.

In a solar cell, electron transport, recombination and lifetime can directly influence the photovoltaic performance. Therefore, electrochemical impedance spectroscopy (EIS) of the pure YS-TiO_2_ and DSL-18 based DSSCs were measured at −0.73 V forward bias in the dark to investigate the behaviors of electron transport and recombination (Fig. 6), and the fitting data results are given in Table 1. From Fig. 6 and Table 1, we can see that, the as-obtained YS-TiO_2_ based DSSC exhibit larger charge transfer and recombination resistance (*R*_ct_) values than DSL-18 nanoparticle based DSSC, suggesting that it has a lower recombination rate than nanoparticle based DSSC. Furthermore, according to the equation: *τ*_*n*(EIS) _= *R*_*ct *_× *C*_*μ*_^21^, the electron lifetime are calculated to be 25.6 and 19.5 ms for YS-TiO_2_ and DSL-18, respectively. Clearly, the as-obtained YS-TiO_2_ based DSSC shows superior electron lifetime compared with the traditional TiO_2_ nanoparticles based DSSC.

**Figure 7:**
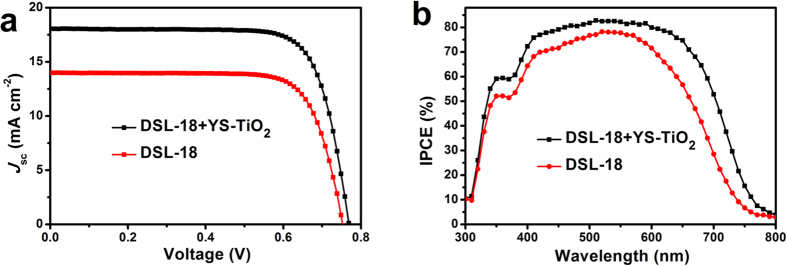
Device performance of DSSCs. *J-V* characteristics (**a**) and Incident photon-to-electron conversion efficiencies (IPCE) (**b**) of the YS-TiO_2_ and DSL-18 based DSSCs.

Owing to the large micrometer size of the as-obtained YS-TiO_2_ microspheres, some light might be back scattered at the FTO-TiO_2_ interface, therefore this part of light usually cannot be utilized by the dye to generate electrons^28^,^30^. Additionally, the connectivity with FTO surface and recombination of generated electrons can greatly influence the performance of a DSSC^31^,^32^. Therefore, in this study, a DSL-18 nanoparticles underlayer and TiCl_4_ post-treatment were employed to improve the performance of YS-TiO_2_ based DSSC^27^,^28^,^33^,^34^, as a result, giving *J*_*sc*_ a high value of 18.84 mA cm^−2^, and consequently resulting in a PCE value up to 11.03% (Fig. 7a, Table 2). For comparison, we also prepared the DSL-18 based photoanode with the same thickness and assembled it to DSSC under the same condition. Whereas, the DSL-18 based DSSC only show a PCE value of 8.01% (Fig. 7a and Table 2). To attest this result, we measured IPCE of both of the DSSCs. From Fig. 7b, we can see that the YS-TiO_2_ microspheres based DSSC exhibits higher IPCE in the wavelength range of 410–800 nm, and especially in the longer wavelength range from 570 to 800 nm. The similar IPCE at shorter wavelengths might be ascribed to similar dye loading capacities of both of the photoanode films, while the higher IPCE at longer wavelength region may be attributed to the superior light harvesting ability induced by light scattering effect of the yolk-shell microspheres^18^,^20^.

## Conclusions

We have demonstrated a one-pot solvothermal approach employing acetylacetone as reaction solvent to synthesis the unique yolk-shell TiO_2_ microspheres. The reaction mechanism was evidenced by FTIR, ^13^CNMR and ESI-MS. By controlling the reaction time, the microspheres diameter and interior space could be tuned. Importantly, the yolk-shell TiO_2_ microspheres were successfully applied as photoanode to construct DSSC. Owing to the high BET surface area and superior light scattering effect, the yolk-shell TiO_2_ microspheres based DSSC exhibit high PCE value up to 11.03%. This work provides a new approach for the synthesis of TiO_2_ microspheres from solid to yolk-shell structure, offering a new materials platform for lithium batteries, catalyst and other applications.

## Methods

### Synthesis of Yolk-shell (YS) TiO_2_ microspheres

All chemicals were purchased from Aldrich and were used as received. Yolk-shell TiO_2_ microspheres were synthesized via a nonaqueous solvothermal process. In a typical synthesis, acetylacetone (acac, 10 ml) was dissolved in 40 ml of isopropyl alcohol under vigorous stirring. Then, tetra-n-butyl titanate (TBT, 2 ml) was rapidly dropped into the solution. After stirring for 5 minutes at ambient condition, the transparent yellowish mixture was transferred to a 100 ml Teflon-lined stainless-steel autoclave. After treatment at 200 °C for 6 h, the yellowish-brown precipitate was collected by centrifugation, washed with ethanol for several times and dried at 60 °C.

### Device fabrication

For the photoanode, single layer films (either DSL-18 or YS-TiO_2_) were screen-printed on FTO-type TCO glass through a 34T meshsize screen. The films were sintered at 510 °C for 30 min before solar cell construction. A 300 μM portion of cheno-3a,7a-dihydroxy-5b-cholic acid was dissolved with an equimolar amount of C101 complex in a mixture of tert-butanol and acetonitrile solvent (1:1 by volume). After being washed by acetonitrile and dried in air, the overnight sensitized electrodes were sealed using a 60 μm thick Surlyn gasket, melted by heating with the Pt-modified TEC15 TCO counter-electrode. The latter was prepared by spreading out a drop of 5 mM H_2_PtCl_6_ isopropyl alcohol solution onto the counter-electrode before treating it at 450 °C for 30 min under air. A hole was introduced in the counter-electrode by sand-blasting, allowing the internal space between the two electrodes to be filled with volatile electrolyte using a vacuum backfilling system, and then was sealed with a thin glass sheet. The electrolyte was composed of 1 M DMII, 50 mM LiI, 30 mM I_2_, 0.5 M tert-butylpyridine, and 0.1 M GuNCS in a solvent mixture of 85% acetonitrile with 15% valeronitrile by volume.

### Characterization

The morphology of the samples was investigated by scanning electron microscopy (FEI XL-30 SFEG coupled to a TLD) and transmission electron microscopy (TEM, JEM-200CX; JEOL). The X-ray diffraction (XRD) patterns were recorded using a Bruker-AXS Microdiffractometer (model D5005) with Cu Kα radiation (λ = 1.5406 Å). The surface area, pore volume and pore size were evaluated by using a micromeritics (TriStar II 3020 V1.03, Micromeritics Instrument Corporation) nitrogen adsorption/desorption apparatus. Ultraviolet-visible (UV–vis) diffuse reflectance spectroscopy and absorption spectroscopy were performed using the UV-vis spectrophotometer (SOLID3700, Shimadzu Co. Ltd, Japan). The product solution obtained after solvothermal reaction was filtered to remove any remaining particles and then analyzed by electrospray ionization mass spectrometry (ESI-MS) (LCQ Fleet), ^13^C liquid-state NMR (Bruker DRX-400), and Fourier Transform Infrared Spectrometer (FTIR) (Thermo fisher IS50R, USA).

The (*J-V*) measurements were carried out on a Keithley model 2420 digital source meter controlled by Test point software. Simulated AM 1.5 illumination was provided by a Newport solar simulator, and light intensity was measured using a calibrated Si solar cell. The active area of the cells was defined by a mask to be 5 × 5 mm^2^. IPCE spectra were measured with a spectral resolution of 5 nm, using a 300 W xenon lamp and a grating monochromator equipped with order sorting filters (Newport/Oriel). The incident photon flux was determined by using a calibrated silicon photodiode (Newport/Oriel). Photocurrents were measured with an auto-ranging current amplifier (Newport/Oriel). Control of the monochromator and recording of the photocurrent spectra were performed with a PC running the TRACQ Basic software (Newport).

## Additional Information

**How to cite this article**: Li, Z.-Q. *et al.* Mesoporous TiO_2_ Yolk-Shell Microspheres for Dye-sensitized Solar Cells with a High Efficiency Exceeding 11%. *Sci. Rep.* **5**, 14178; doi: 10.1038/srep14178 (2015).

## Supplementary Material

Supplementary Information

## Figures and Tables

**Table 1:** EIS parameters.

Cell	*R*_ct_ (Ω)	*C*_μ_ (*μ*F)	*τ*_n(EIS)_ (ms)
**YS-TiO**_**2**_	33.1	771.49	25.6
**DSL-18**	24.5	796.11	19.5

Electron transfer and recombination resistance (*R*_ct_) and chemical capacitance (*C*_*μ*_) of YS-TiO_2_ and DSL-18 based DSSCs

**Table 2:** Photovoltaic parameters.

Cell	Thickness	*J*_sc_ (mA cm^−2^)	*V*_*oc*_ (V)	FF (%)	*η* (%)
**DSL-18+YS-TiO**_**2**_	4.5 *μ*m + 7.1 *μ*m	18.84	0.769	76.10	11.03
**DSL-18**	11.5 *μ*m	14.02	0.753	75.92	8.01

Comparison of the photovoltaic properties measured under 1 sun illumination and for YS-TiO_2_ and DSL-18 based DSSCs
