# Supplementary material for: Mesoporous TiO2 Yolk-Shell Microspheres for Dye-sensitized Solar Cells with a High Efficiency Exceeding 11%
Source: Sci Rep. 2015 Sep 18;5:14178. doi: 10.1038/srep14178 (PMC4585661; doi:10.1038/srep14178)
Supplement: Supplementary Information [file srep14178-s1.doc]

**Supplementary Information**

**Mesoporous TiO_2_ Yolk-Shell Microspheres for Dye-sensitized Solar Cells with a High Efficiency Exceeding 11%**

Zhao-Qian Li^1^, Wang-Chao Chen^1^, Fu-Ling Guo^1^, Li-E Mo^1^, Lin-Hua Hu^1^, & Song-Yuan Dai^2,1^

^1^Key Laboratory of Novel Thin-Film Solar Cells, Institute of Applied Technology, Hefei Institutes of Physical Science, Chinese Academy of Sciences, Hefei, Anhui, 230031, P. R. China.

^2^Beijing Key Laboratory of Novel Thin-Film Solar Cells, North China Electric Power University, Beijing, 102206, P. R. China.

Correspondence and requests for materials should be addressed to L.-H.H. (solarhu@sina.com) or S.-Y.D. (sydai@ipp.cas.cn)


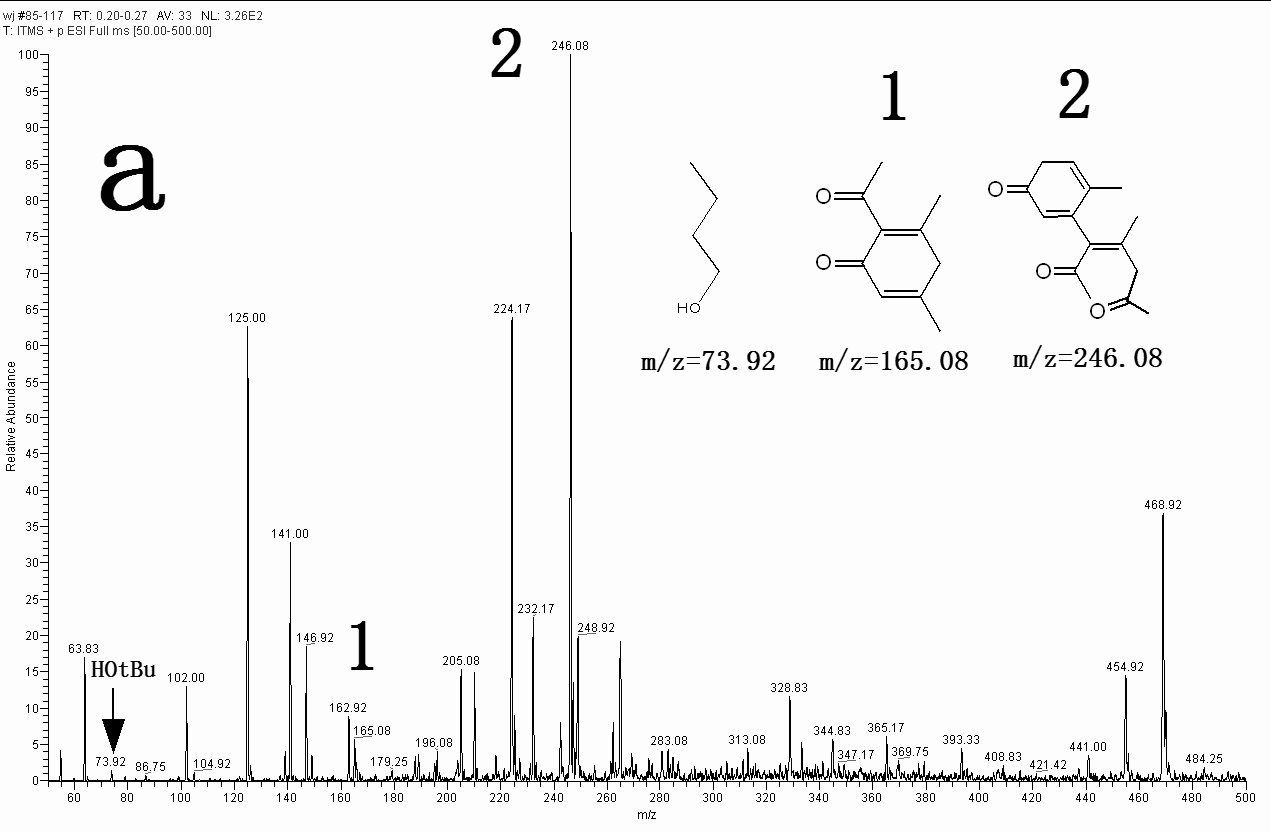


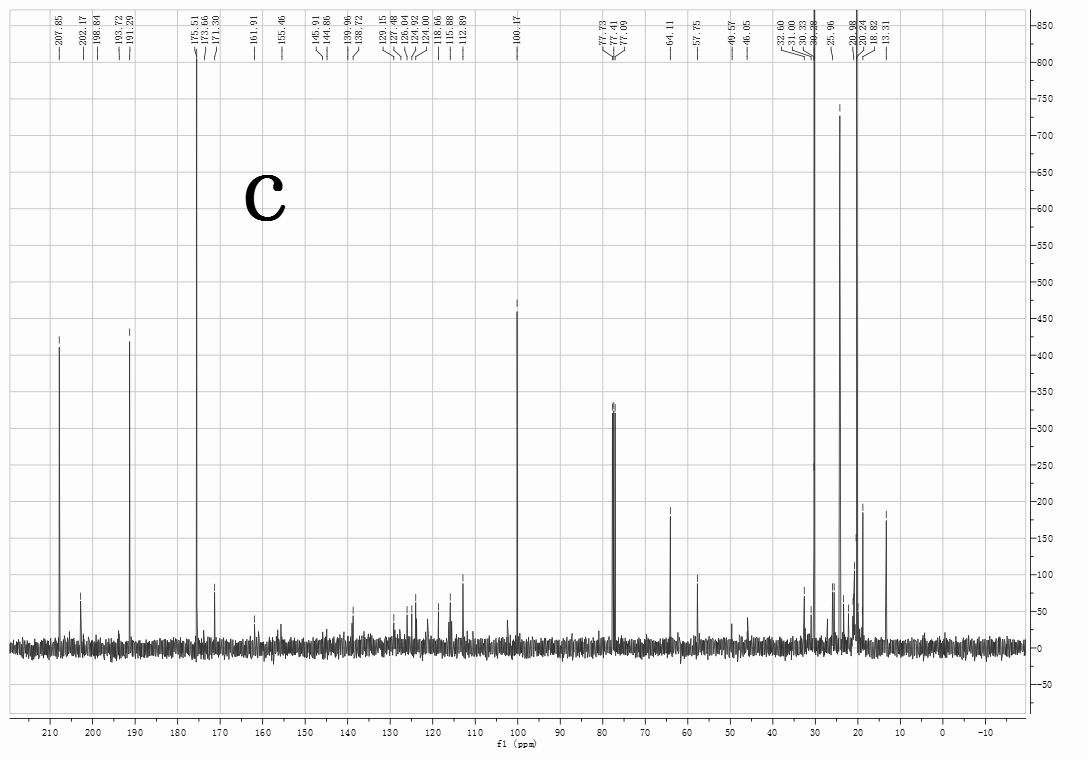


**Figure S1∣ESI-MAS, FTIR and ^13^CNMR.** (a) ESI-MAS, (b) FTIR and (c) ^13^CNMR indicating the reaction of acetylacetone in the presence of tetra-n-butyl titanate. Formation of C=C and H_2_O can evidence the aldol condensation of acetylacetone because there is no existence of C=C in isopropyl alcohol, acetylacetone and tetra-n-butyl titanate.

**^13^CNMR data:**

**Product 1:**

*2-acetyl-3,5-dimethylcyclohexa-2,5-dienone****:***

^13^CNMR δ = 145.9 (C1), 127.4 (C2), 191.3 (C3), 46.1 (C4), 139.9 (C5), 161.9 (C6), 198.8 (C7).

m/z = 165.08

**Product 2:**

*4-methyl-3-(6-methyl-3-oxocyclohexa-1,5-dien-1-yl)hept-3-ene-2,6-dione:*

^13^CNMR δ = 207.8 (C1), 198.8 (C2), 139.9 (C3), 138.7 (C4), 155.4 (C5), 129.1 (C6), 191.3 (C7), 138.7 (C8).

m/z = 246.08


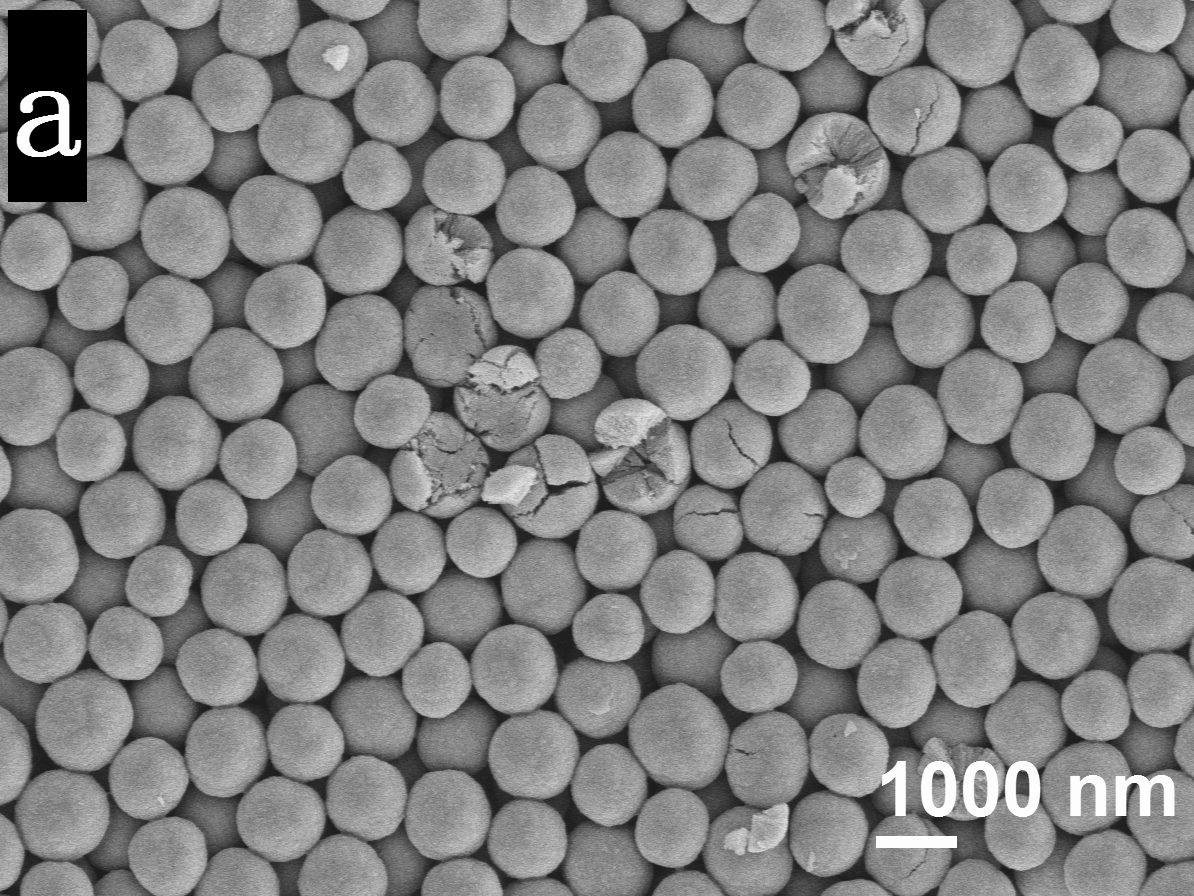

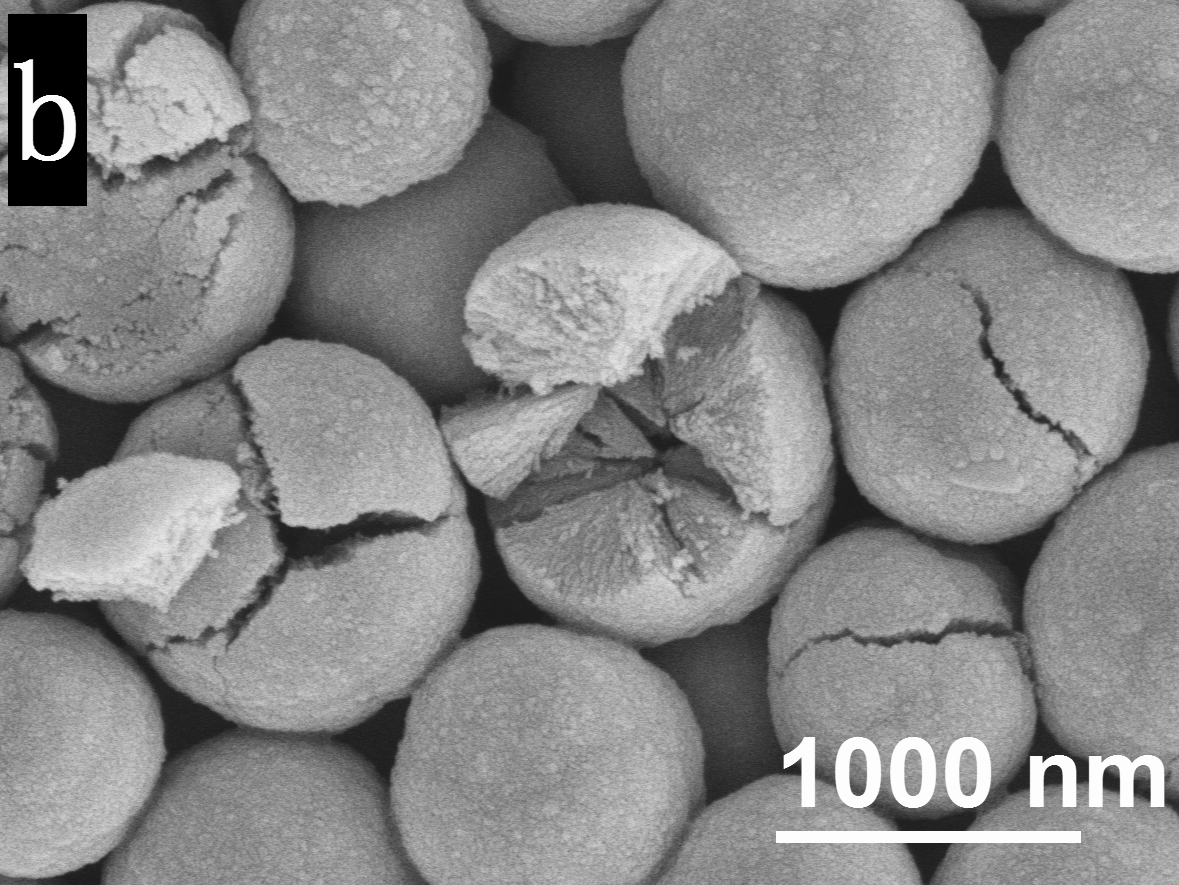


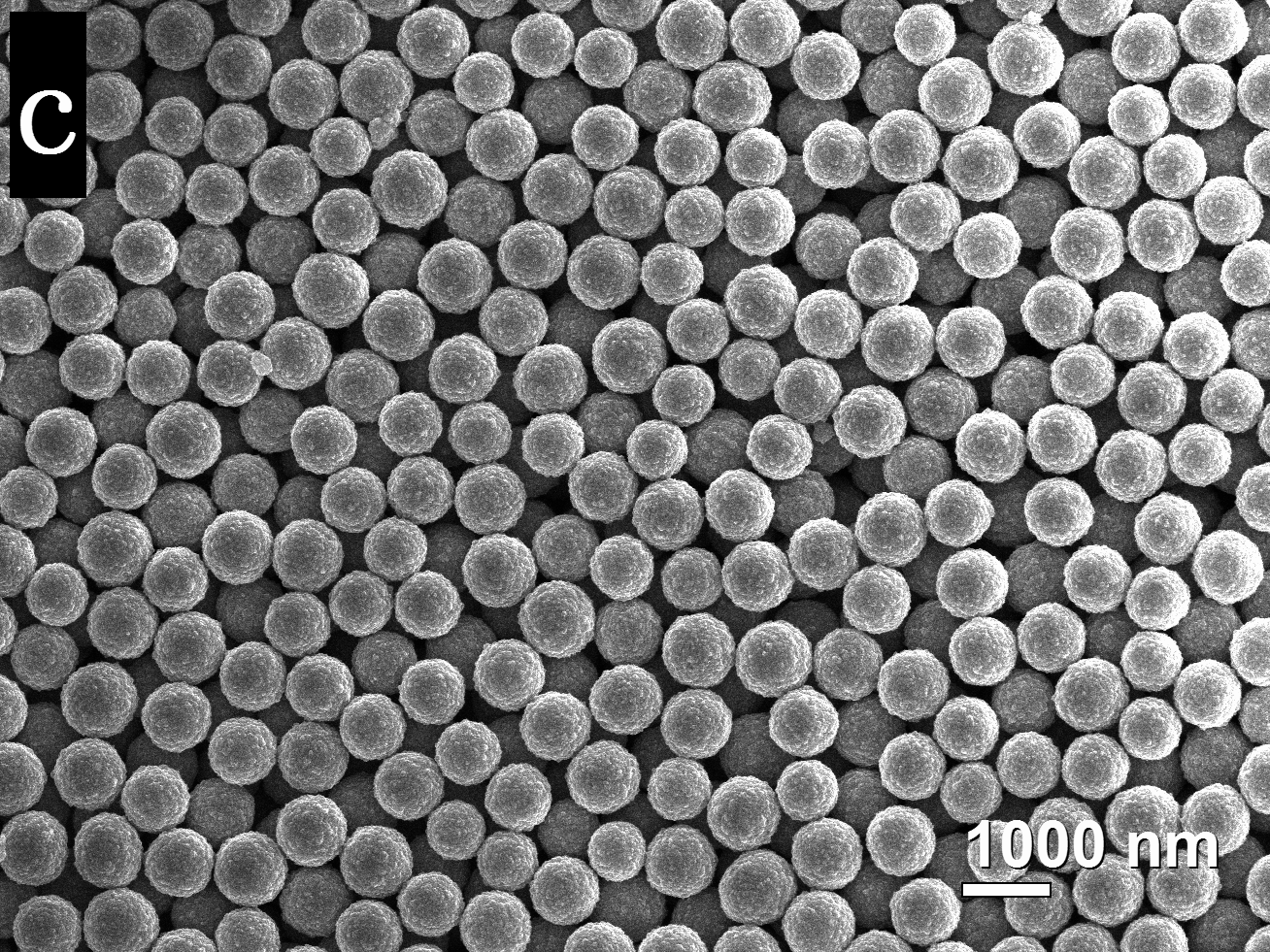

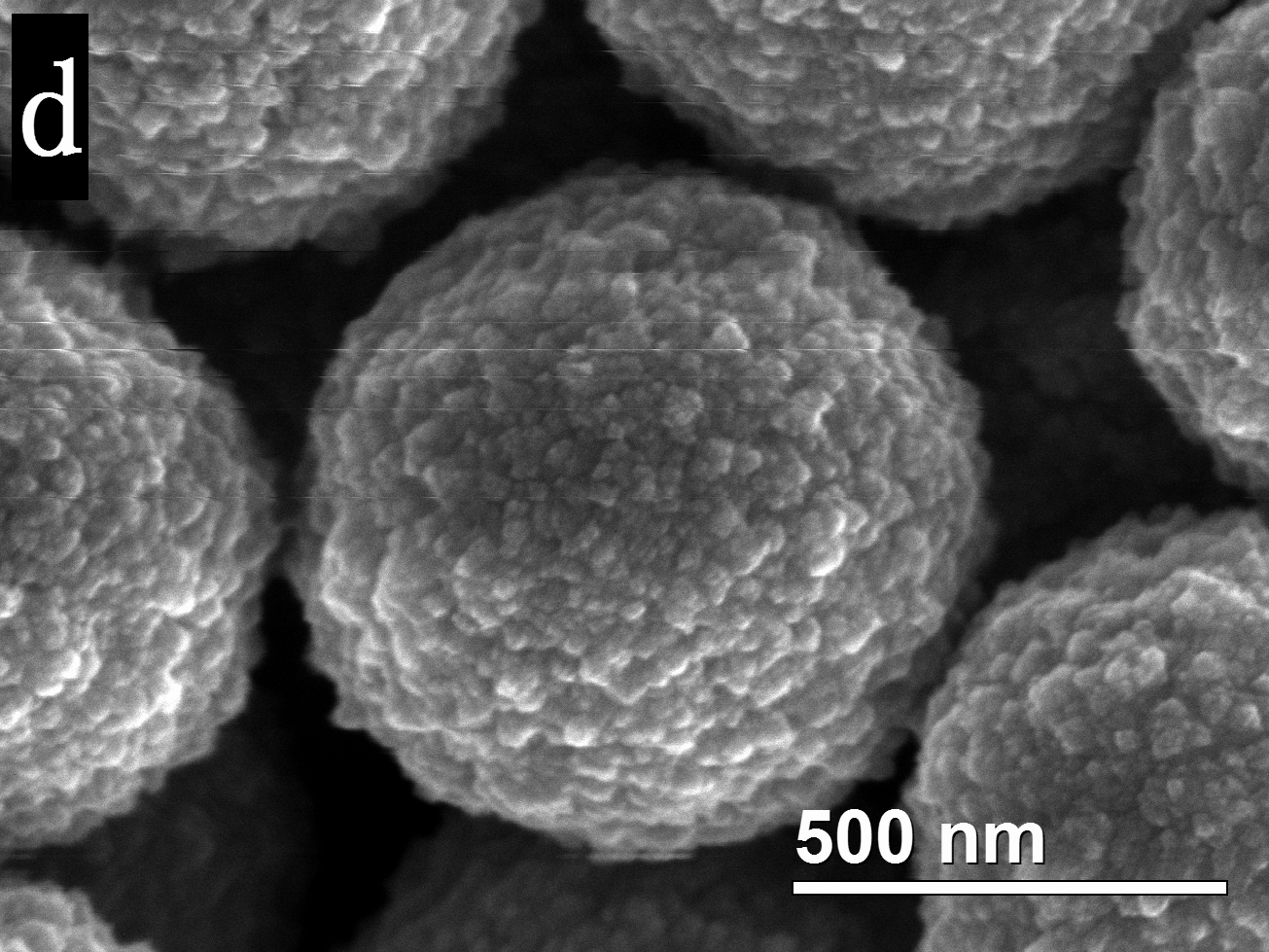


**Figure S2∣SEM images.** SEM images of the TiO_2_ microspheres synthesized at 200 °C for 6 h without addition of isopropyl alcohol (only 50 ml of acac) (a, b) and with 25 ml of IPA and 25 ml of acac (c, d).
